# Evaluating YOLO26s for Multi-Class Pavement Crack Detection: A Lightweight Approach for Sustainable Edge Deployment

**DOI:** 10.3390/s26165113

**Published:** 2026-08-12

**Authors:** Saifal Abbas, Md Taherul Islam Shawon, Saqib Qamar, Muhammad Adeel

**Affiliations:** 1 School of Highway, Chang’an University, Xi’an 710064, China; 2024021903@chd.edu.cn (S.A.); 2024121911@chd.edu.cn (M.T.I.S.); 2 Department of Intelligent Systems, KTH Royal Institute of Technology, 10044 Stockholm, Sweden; 3 Faculty of Computing and IT (FCIT), Sohar University, Sohar 311, Oman; 4 School of Civil Engineering and Architecture, Wuhan University of Technology, Wuhan 430070, China; muhammadadeel@whut.edu.cn

**Keywords:** sustainable infrastructure, pavement crack detection, YOLO, multi-class classification, road maintenance, deep learning

## Abstract

**Highlights:**

**What are the main findings?**

- YOLO26s, a lightweight deep learning model, achieves 89.0% mAP@0.5 for multi-class pavement crack detection (longitudinal, transverse, pothole, and alligator cracks) while reducing 14.3% parameters and 7.7% FLOPs compared to YOLOv8s, enabling efficient real-time edge deployment.
- Evaluated on a multinational dataset (RDD2022) spanning four countries (USA, Norway, Japan, China), YOLO26s demonstrates robust cross-domain generalization across diverse environmental conditions, road textures, and imaging perspectives.

**What are the implications of the main findings?**

- The lightweight architecture supports sustainable infrastructure management by facilitating early, accurate crack detection on resource-constrained edge devices, reducing material waste, energy consumption, and carbon emissions from delayed or repeated road maintenance.
- The model’s real-time capability and computational efficiency make it scalable for large-scale road network monitoring, offering a practical AI-driven solution for municipalities and maintenance authorities in both developed and emerging regions.

**Abstract:**

Maintaining durable road infrastructure is crucial for reducing resource consumption, minimizing repair costs, and supporting sustainable urban mobility. However, accurately detecting small and morphologically diverse pavement cracks remains challenging due to variations in lighting, road textures, and crack shapes across different geographic regions. YOLO (You Only Look Once) is one of the most widely adopted deep learning (DL) frameworks for object detection. Traditional inspection methods are labor-intensive and often inconsistent, while existing DL models can be computationally heavy or limited to single crack types, restricting real-time deployment and scalability. To address these challenges, this study presents YOLO26s, a lightweight DL model for multi-class pavement crack detection across diverse environmental and geographic conditions. Using a curated subset of 6972 annotated images from the Road Damage Dataset 2022, YOLO26s identifies four crack types: longitudinal, transverse, pothole, and alligator cracks. Compared to baseline models (YOLOv8s, YOLOv8n, YOLO26n), YOLO26s achieves higher detection accuracy (mAP@0.5 = 89.0%) while reducing computational complexity by 14.3% in parameters and 7.7% in FLOPs, enabling real-time deployment on edge devices. By facilitating early and accurate crack detection, the proposed approach supports proactive maintenance, extends pavement lifespan, and reduces material and energy usage, contributing to more sustainable road network management. These findings highlight the potential of efficient AI-driven inspection systems to enhance environmental and economic sustainability in civil infrastructure.

## 1. Introduction

Road infrastructure is a fundamental pillar of urban mobility, economic development, and societal safety [1,2]. Well-maintained pavements facilitate efficient movement of people and goods while reducing vehicle operating costs and accident risks. Pavement deterioration, manifesting as longitudinal, transverse, pothole, and alligator cracks, directly impacts both operational efficiency and maintenance costs. Delayed detection of such cracks often leads to increased material consumption, energy expenditure, and environmental emissions associated with repair operations, highlighting the environmental and economic stakes of traditional maintenance strategies [3,4]. The American Society of Civil Engineers (ASCE) estimates that poor road conditions in the United States alone result in over USD 130 billion annually in additional vehicle operating costs and travel delays [5]. This economic and environmental burden underscores the importance of implementing efficient, scalable, and proactive pavement monitoring strategies. Traditionally, pavement condition assessments relied on manual visual inspections conducted by trained engineers [6,7]. While effective for small-scale surveys, this approach is labor-intensive, subjective, and inconsistent across inspectors. The process is also difficult to scale across urban and intercity networks spanning thousands of kilometers, often delaying critical maintenance decisions and increasing both economic and environmental costs [8]. To address these challenges, early automation efforts applied classical computer vision techniques such as thresholding, edge detection, texture analysis, and handcrafted feature extraction [9,10]. These approaches reduced labor intensity but struggled under heterogeneous real-world conditions characterized by variations in lighting, pavement texture, shadowing, occlusions, and diverse crack morphologies.

The emergence of DL, particularly convolutional neural networks (CNNs), significantly advanced automated pavement crack detection by learning hierarchical features directly from images. Single-stage object detectors [11,12] such as the YOLO family of object detectors (YOLOv8 to YOLOv26) have evolved with improved backbones, attention mechanisms, and enhanced detection heads, enabling accurate recognition of small and complex targets [13,14]. YOLO-based detectors have similarly been applied to surface-defect inspection in other infrastructure and manufacturing domains, such as precision metal workpiece inspection under variable lighting and surface texture [15,16], underscoring the architecture’s suitability for small, low-contrast target detection beyond its original benchmark domain. Enhanced YOLO architecture pipelines integrated with multi-frame tracking or attention mechanisms have achieved high accuracy in complex environments. This study introduces a lightweight YOLO framework for concrete crack detection and segmentation, emphasizing edge-deployment feasibility [17]. These advances can improve multi-class pavement crack detection, supporting real-time, automated, and scalable road maintenance systems [18,19]. Despite these advances, three application-specific gaps limit the practical deployment of YOLO-based crack detection in multinational road network monitoring programs. First, models validated on single-country datasets risk performance degradation when deployed across the pavement textures, camera perspectives, and lighting conditions of other regions [20], a direct obstacle for any agency operating across multiple jurisdictions. Second, models restricted to single-class crack detection cannot support maintenance programs that prioritize repairs by damage type, since longitudinal, transverse, alligator, and pothole damage call for different repair methods and indicate different underlying failure mechanisms [21]. Third, prior benchmarking has generally reported accuracy without reporting the computational cost of achieving it, leaving practitioners unable to judge deployability on resource-constrained edge hardware [22]. These factors are critical for sustainable and scalable deployment on edge devices in urban and rural road networks.

To address these gaps, the Road Damage Dataset 2022 (RDD2022) provides a multinational benchmark of over 47,000 annotated images collected from multiple countries, capturing diverse road textures, environmental conditions, and lighting scenarios [23]. This dataset enables rigorous evaluation of DL models under realistic and heterogeneous conditions, facilitating the development of solutions that generalize effectively across regions. Nevertheless, systematic benchmarking of lightweight YOLO-based architectures, particularly YOLO26 variants, on multi-class crack detection tasks using this dataset remains absent in the literature [24]. This study systematically evaluates YOLO26s [25], a lightweight YOLO architecture, for multi-class pavement crack detection in heterogeneous environments, assessing whether it balances high detection accuracy with computational efficiency well enough for real-time edge deployment. YOLO26s incorporates compact backbone modules, attention-guided feature selection, and efficient bounding-box regression to enable real-time edge deployment, facilitating proactive maintenance and sustainable infrastructure management. Early detection and classification of cracks using YOLO26s allow timely repair scheduling, reducing material use, energy consumption, and carbon emissions associated with repeated or delayed maintenance cycles [26]. By prioritizing lightweight design and edge-compatible deployment, the model supports environmentally and economically sustainable road network operations, particularly in resource-constrained settings or emerging regions where high-performance computational infrastructure may be limited.

The primary contributions of this work are fourfold:

- Proposal and evaluation of YOLO26s on a 6972-image subset of RDD2022 to capture diverse pavement textures, lighting, and imaging conditions.
- Comparative benchmarking against YOLOv8s, YOLOv8n, and YOLO26n to assess multi-class crack detection performance, computational efficiency, and suitability for real-time edge deployment.
- Detailed analysis of training dynamics, precision–recall metrics, F1 scores, and confusion matrices, providing insights for reproducibility and practical deployment decisions.
- Demonstration that YOLO26s achieves high detection performance (mAP@0.5 = 89%) while reducing parameters and FLOPs compared to baseline architectures, offering an efficient solution for sustainable, real-time pavement monitoring.

Through these contributions, this work not only advances the technical capabilities of automated crack detection but also provides a foundation for sustainable infrastructure management, reducing material waste, operational energy use, and the environmental footprint of road maintenance programs. Furthermore, by demonstrating the feasibility of edge deployment, YOLO26s enables scalable, real-time monitoring of extensive road networks, supporting proactive decision-making for infrastructure agencies, municipalities, and road maintenance authorities.

The remainder of this manuscript is organized as follows. Section 2 describes the methods, including dataset preparation, annotation, data augmentation, model architecture, training, and loss functions. Section 3 presents experimental results on quantitative metrics, complexity, efficiency, convergence, confidence, confusion matrix, and qualitative inspection. Section 4 discusses YOLO26s performance relative to state-of-the-art methods, cross-architecture comparisons, and implications for generalization and sustainable infrastructure, along with limitations and future edge-deployment directions. Section 5 concludes with key findings.

## 2. Methods

### 2.1. Dataset Overview and Preparation

The dataset used in this study is a curated subset of the Road Damage Dataset 2022 (RDD2022) [23], a publicly available multinational benchmark containing 47,420 road inspection images from six countries. The India and Czech Republic subsets, originating from the earlier RDD2020 stream, were not included in this curated export because they retained the PASCAL-VOC XML schema and were not available in the uniformly processed YOLO-format release used here. The subset used here consists of 6972 images from four countries: The United States, Norway, Japan, and China. The images were collected using dashcam and motorbike-mounted cameras under diverse road conditions, including urban streets, rural highways, mountainous routes, and residential areas. This geographic and environmental diversity ensures robust evaluation of model generalization across substantially different pavement materials, crack morphologies, and imaging perspectives. All images are annotated in YOLO bounding-box format with axis-aligned bounding boxes [27]. Four damage categories are defined, following the structural damage taxonomy of the RDD2022 annotation protocol as described in Table 1.

In Table 1, each row lists the class ID, category, and a concise description of the damage type. Longitudinal cracks result from traffic loading, transverse cracks from thermal effects, potholes from aging and material volume changes, and alligator cracks indicate structural fatigue. The table provides clear semantic definitions to ensure reproducible annotation and consistent evaluation across models. The dataset was partitioned into training (70%), validation (20%), and testing (10%), resulting in 4880 training images, 1394 validation images, and 698 test images. This split ensures sufficient data for both model learning and unbiased evaluation. To enhance model robustness and prevent overfitting, we applied a comprehensive set of online data augmentation techniques:

1. Random horizontal and vertical flips: These flips ensure the model becomes invariant to orientation and road layout. The image transformation, including horizontal and vertical flips, where W and H are the width and height of the image, respectively are as follows:

(1) $$I^{\prime }\left(x,y\right)=I(W-x,y)$$

(2) $$I^{\prime }\left(x,y\right)=I(x,H-y)$$

2. Random scaling within ±50%: Images are scaled to simulate variations in camera distance, ensuring the model can detect cracks at multiple scales $x^{\prime }=S_{x}\cdot x$ and $y^{\prime }=S_{y}\cdot y$.
3. Mosaic augmentation: Combines four images into one, providing diverse contextual backgrounds and small-object examples, essential for cracks that occupy a tiny portion of the image.
4. HSV (Hue, Saturation, Value) jittering: Simulates varying illumination conditions to improve robustness under sunlight, shadow, and nighttime conditions.
5. Random rotation within ±10°: Accounts for variations in camera orientation and road alignment.
6. Copy-paste augmentation: Augments underrepresented classes by duplicating small cracks and pasting them onto different backgrounds, balancing class distribution.

**Figure 1:**
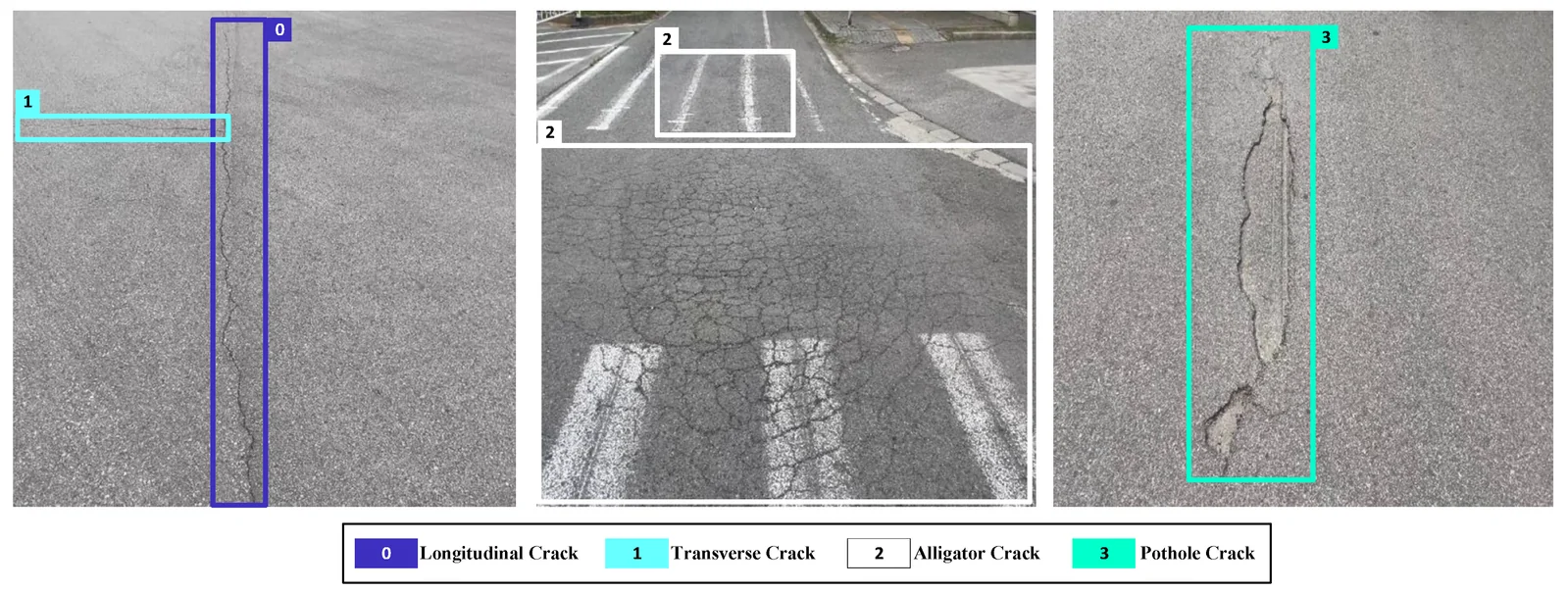
Representative annotated images for each of the four RDD2022 crack categories evaluated in this study. Bounding boxes are color-coded by class, as indicated in the legend.

Figure 1 presents representative images from the curated subset of RDD2022 used for training and evaluation. Each image contains bounding-box annotations for the four crack types: longitudinal cracks (Class 0), transverse cracks (Class 1), potholes (Class 2), and alligator cracks (Class 3). Longitudinal cracks are typically thin and aligned with the lane direction, while transverse cracks appear perpendicular and vary in width. Potholes form rectangular networks dividing the surface into potholes, and alligator cracks exhibit interconnected, fatigue-like patterns. The dataset includes diverse roadway scenes captured under varying pavement textures, lighting conditions, camera perspectives, and geographic environments. The annotations illustrate the complexity of real-world road damage detection, including thin low-contrast cracks, irregular crack patterns, and large-area pavement distress regions.

**Figure 2:**
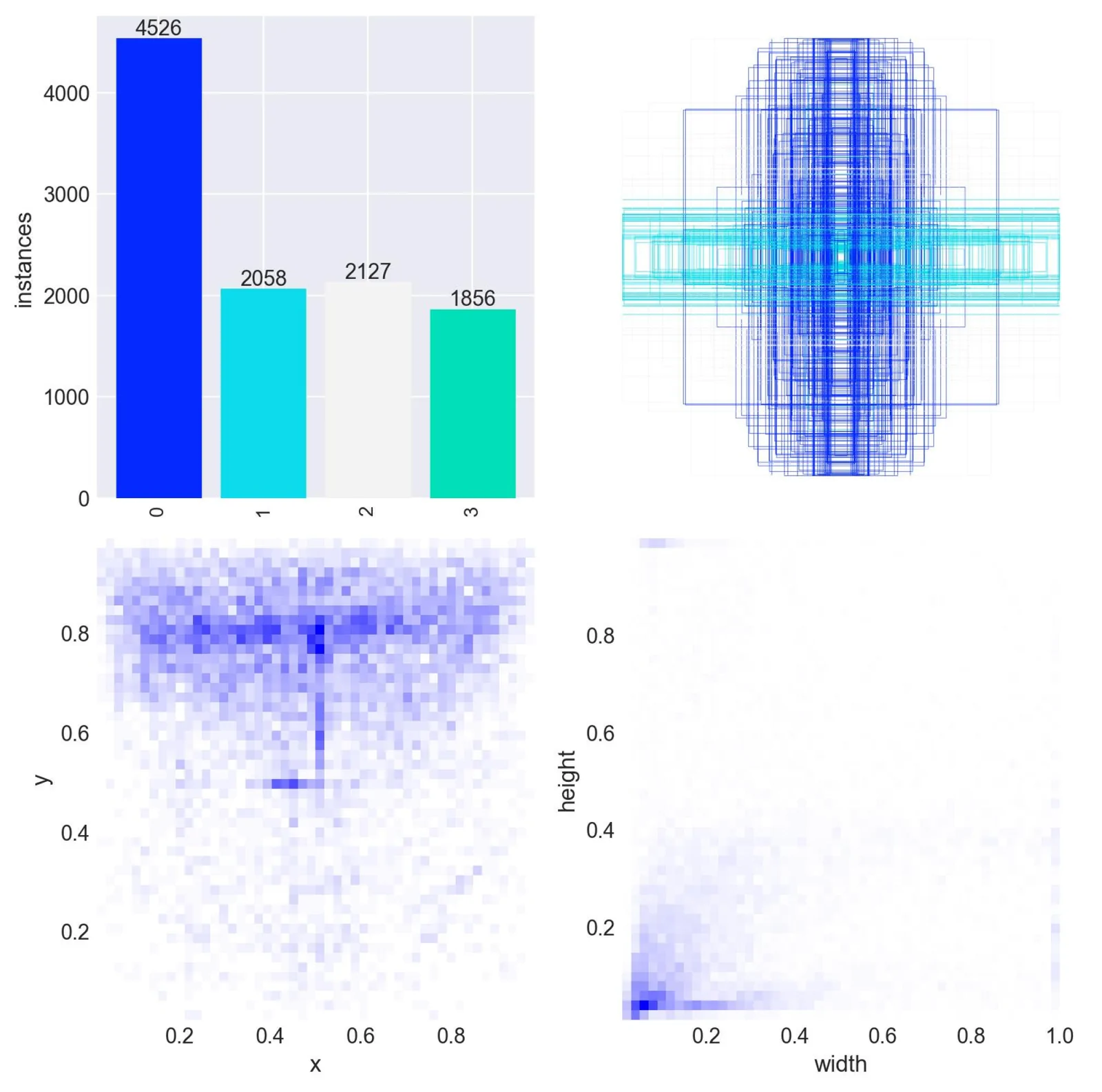
Class distribution and bounding-box statistics for the curated 6972-image RDD2022 subset. **Top-left**: number of annotated instances per class. **Top-right**: normalized spatial distribution of bounding-box centers. **Bottom-left**: kernel-density heatmap of bounding-box centre locations. **Bottom-right**: joint distribution of bounding-box width and height.

Figure 2 visualizes the class distribution and bounding-box spatial statistics, including heatmaps of crack locations, bounding-box size distributions, and overlap statistics, highlighting the variability in crack morphology and scale. In Figure 2, the top-left chart displays the number of annotations per class, revealing class imbalance, with longitudinal cracks being more common than pothole or alligator cracks. The top-right plot shows the spatial distribution of bounding-box centers, normalized to image coordinates, indicating how cracks appear across the road frame. The bottom-left heatmap depicts the density of bounding-box center locations, highlighting areas of frequent crack occurrence, while the bottom-right heatmap illustrates bounding-box width and height distributions, reflecting variations in crack size. These statistics demonstrate the dataset’s heterogeneity, which is crucial for training models that generalize across geographic and environmental variations.

This dataset design ensures that the model is trained under heterogeneous and realistic conditions, which is crucial for sustainable deployment. By improving detection generalization, maintenance operations can be automated efficiently, reducing unnecessary inspections, vehicle fuel usage, and material waste.

### 2.2. Model Architecture

YOLO formulates object detection as a single-stage regression problem [28]. Given an input image I of spatial dimensions H×W×3, the network produces bounding-box predictions and class probability scores in a single forward pass. YOLO26s is a lightweight [29], high-accuracy object detection model specifically designed for multi-class pavement crack detection. It builds upon YOLOv8 but introduces three key modifications for accuracy–efficiency optimization:

#### 2.2.1. Compact Backbone Blocks

YOLO26 replaces YOLOv8′s C2f backbone modules with C3k2 compact bottleneck blocks, reducing floating-point operations (FLOPs) while preserving gradient flow and feature extraction capabilities [30]. In deeper layers, the kernel size can optionally be set to 3 × 3 to improve representation without significantly increasing computation. This architectural choice supports edge deployment on low-power hardware while maintaining multi-scale feature extraction.

#### 2.2.2. Spatial Attention Module

The C2PSA (Cross-Stage Partial with Spatial Attention) module is inserted after the SPPF layer [31]. It allows the backbone to selectively emphasize crack-relevant regions while suppressing irrelevant background. The multi-head self-attention mechanism is defined as:

(3) $$Attn\left(Q,K,V\right)=softmax\left(\frac{{QK}^{⊺}}{\sqrt{d_{k}}}\right)V$$

(4) $$Y=Norm(X+W_{o}\;\cdot \;Attn(W_{Q}X,\;W_{K}X,\;W_{V}X))$$

where $d_{k}$ is the key dimension and $W_{Q}$, $W_{K}$, $W_{V}$, and $W_{o}$ are learned projection matrices. This mechanism improves spatial localization, particularly for thin or low-contrast cracks, and enhances model robustness across geographically diverse datasets.

#### 2.2.3. Regression Loss Without DFL

Unlike YOLOv8, which represents each bounding-box edge as a discrete probability distribution over 16 bins and applies Distribution Focal Loss (DFL) to it [32,33], YOLO26 sets the distribution bin count to one (${reg}_{max}=1$), which removes the distributional regression head entirely. Ultralytics’ official documentation for YOLO26 describes this as full DFL removal, undertaken to simplify the detection head and improve export/hardware compatibility [24]. In its place, the box-regression head is supervised with a direct L1 loss on the left/top/right/bottom edge distances, normalized by stride and image size. Because this replacement loss operates on normalized targets in a far smaller numeric range than the 16-bin cross-entropy DFL it replaces, it is substantially smaller in magnitude, which is why the training logs in Table 8 report a value of approximately 0.01 for YOLO26 versus approximately 1.2 for YOLOv8 in the column labeled ‘DFL’: both frameworks retain this column name for backward-compatible logging, but for YOLO26 the value reported is this normalized L1 term rather than a true distributional loss.

#### 2.2.4. Anchor-Free Detection

YOLO26s uses an anchor-free detection head, decoding raw outputs $t_{x}$, $t_{y}$, $t_{w}$, $t_{h}$ are decoded as follows:

(5) $$b_{x}\;=\;(c_{x}\;+\;\sigma (t_{x}))\;\times \;s\;$$

(6) $$b_{y}=(c_{y}+\sigma (t_{y}))\times s$$

(7) $$b_{w}=exp(t_{w})\times s$$

(8) $$b_{h}=exp(t_{h})\times s$$

where $b_{x}$ and $b_{y}$, predicted center coordinates of the bounding box in pixels, while $b_{w}$ and $b_{h}$ predicted width and height of the bounding box in pixels. $c_{x}$ and $c_{y}$ are the grid cell coordinates. YOLO divides the image into a grid (e.g., 20 × 20). Each grid cell predicts object offsets relative to its top-left corner. σ(⋅) is the sigmoid activation function, which maps the raw network output to a range between 0 and 1. Adding the sigmoid-transformed offset $\sigma (t_{x})$ or $\sigma (t_{y})$ gives the absolute position of the box center inside that cell. This ensures the predicted offset stays inside the grid cell [34]. For example, a sigmoid output of 0.5 means the box center is halfway across the cell.

The confidence score and class probabilities are combined to produce the final detection score:

(9) $${Confidence}_{i}\;=\;P(object)\;\times \;IoU(pred,\;gt)\;$$

(10) $${Score}_{i}=P({class}_{i}\;|\;object)\times {Confidence}_{i}$$

**Figure 3:**
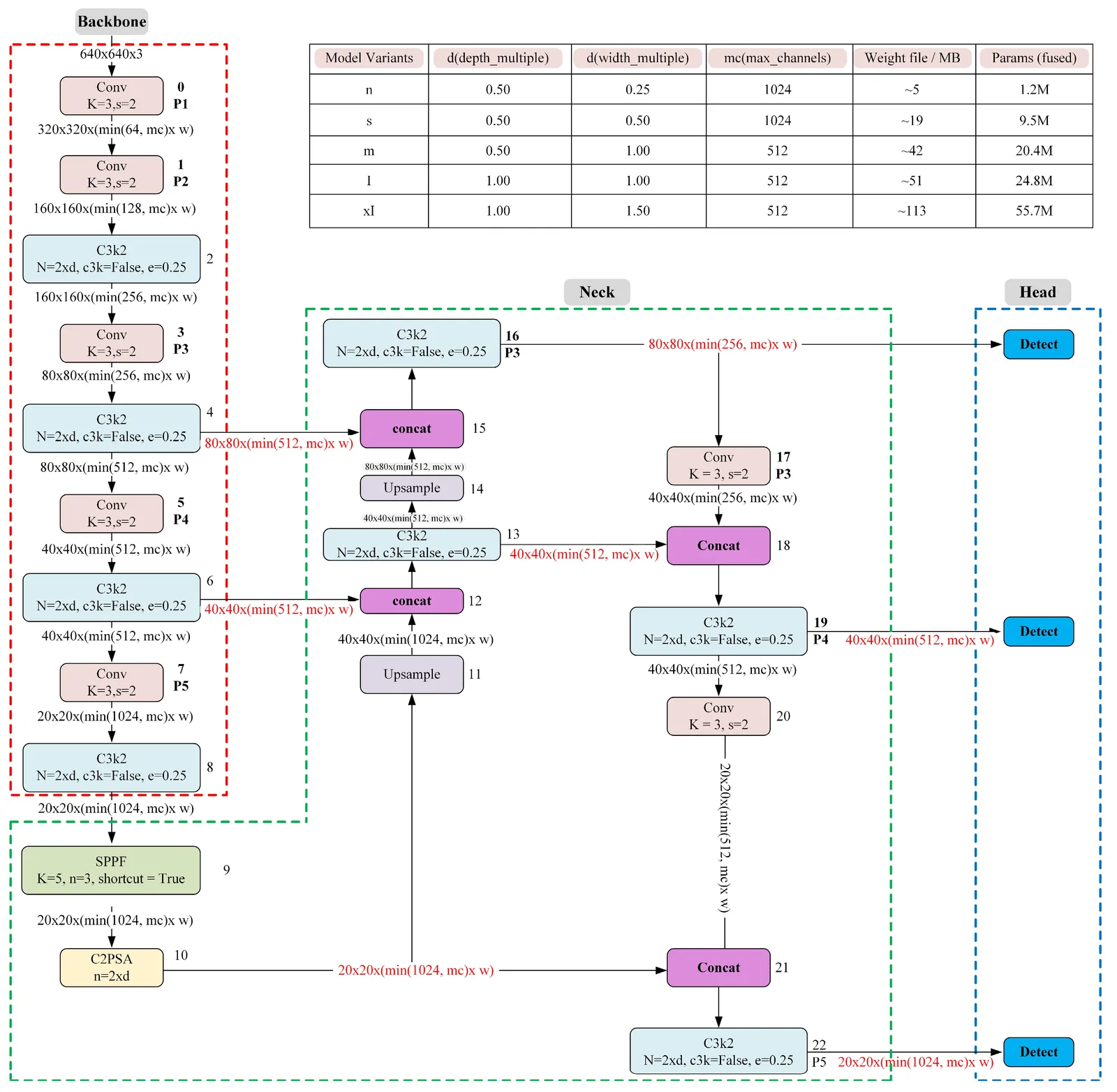
YOLO26 architecture, showing the C3k2 compact backbone blocks, the C2PSA spatial-attention module inserted after SPPF, and the decoupled anchor-free detection head.

Figure 3 illustrates the architecture of YOLO26s, including the C3k2 compact backbone blocks, C2PSA attention module, and decoupled anchor-free detection head. The backbone extracts multi-scale features from input images, with C3k2 blocks reducing FLOPs while maintaining feature richness. The C2PSA module applies spatial attention to emphasize crack-relevant regions, suppressing background noise from varied road textures and lighting. The neck fuses features at different scales, and the detection head predicts bounding boxes, class scores, and confidence in a single forward pass. The figure shows the flow from input image through backbone, attention-guided feature fusion, and final detection, visually explaining the enhancements introduced in YOLO26s relative to YOLOv8. Both YOLOv8 and YOLO26 use Task-Aligned Assignment (TAL) to dynamically match ground-truth boxes to anchor points during training. TAL computes an alignment score that jointly considers classification quality and localization quality:

(11) $$t_{i}=s_{i}^{\alpha }\cdot u_{i}^{\beta }$$

where $s_{i}$ is the predicted classification score and $u_{i}=IoU({\hat{b}}_{i},b_{gt})$ is the predicted Intersection over Union (IoU) quality. Hyperparameters α = 0.5 and β = 6.0 weight the contribution of classification versus localization. The top-k anchor points with the highest $t_{i}$ are assigned as positive samples for each ground-truth box.

### 2.3. Baseline Models

Three baseline models are used for comparative evaluation against the proposed YOLO26s. Table 2 lists the hyperparameters used to train YOLO26s and baseline models under identical conditions (Table S1 with four additional baseline models). It includes input resolution (640 × 640 px), number of epochs (100), batch size (16), optimizer (SGD with momentum 0.937), learning rate schedule (cosine annealing from 1 × 10^−3^ to 1 × 10^−5^), warmup epochs (3), weight decay (5 × 10^−4^), and loss component weights (7.5/0.5/1.5). These parameters were selected to ensure stable convergence, fair comparison across architectures, and reproducibility. The table supports transparency in training configuration, which is critical for real-world deployment and sustainability considerations.

**Figure 4:**
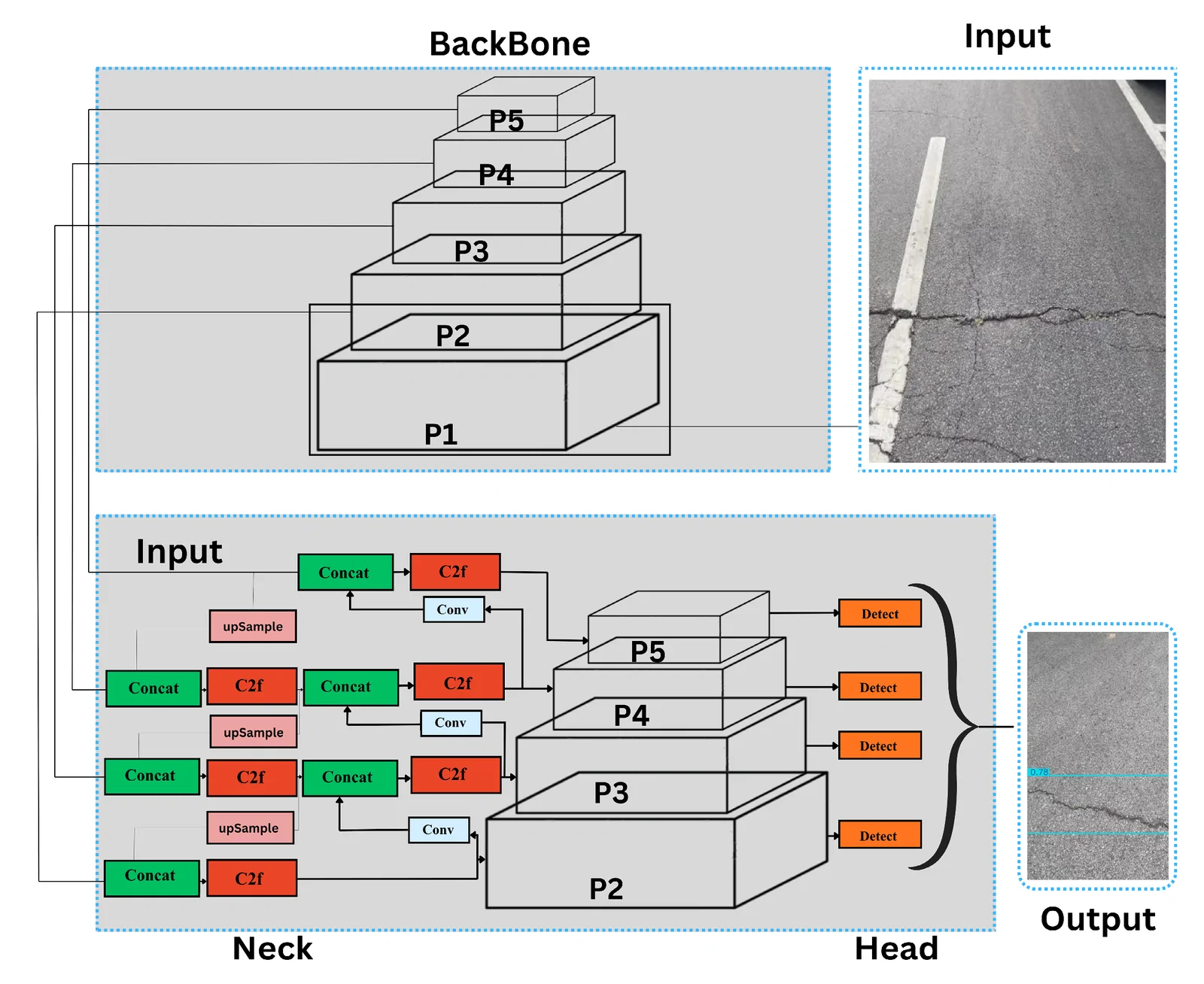
Side-by-side architectural comparison of YOLOv8 and YOLO26s, highlighting the replacement of C2f blocks with C3k2 blocks and the addition of the C2PSA attention module in YOLO26s.

YOLOv8s is the small-scale variant of YOLOv8. It introduces a CSPNet backbone, a decoupled anchor-free detection head, C2f (Cross-Stage Partial with two Bottlenecks) modules, SPPF (Spatial Pyramid Pooling Fast), and a PAN-FPN neck as depicted in Figure 4. With 11.2M parameters and 28.6 GFLOPs, YOLOv8s serves as the primary scale-equivalent baseline for YOLO26s.

YOLOv8n is the nano-scale variant of YOLOv8 (3.2M parameters, 8.7 GFLOPs) and represents the most compact member of the YOLOv8 family evaluated in this study.

YOLO26n is the nano-scale variant of YOLO26 (2.6M parameters, 6.9 GFLOPs). It is the lightest model evaluated, included to quantify the intra-family accuracy–efficiency trade-off within the YOLO26 architecture.

Figure 4 compares the original YOLOv8 small-scale architecture (YOLOv8s) with the proposed YOLO26s. The backbone, neck, and detection head stages are shown side by side, highlighting key differences: YOLO26s replaces C2f blocks with C3k2 bottlenecks and introduces the C2PSA attention module after SPPF. Both models use decoupled detection heads; however, YOLO26s adds stride-normalized DFL for improved bounding-box localization. The figure provides a visual map of module replacements and enhancements [35,36], explaining why YOLO26s achieves higher accuracy with fewer parameters and FLOPs.

### 2.4. Loss Functions

The total training loss combines bounding-box regression, classification, and DFL:

(12) $$L_{total}=\lambda_{box}L_{box}+\lambda_{cls}L_{cls}+\lambda_{dfl}L_{dfl}$$

where $L_{total}$ is the overall loss used to train YOLO26s. It combines three separate losses: bounding-box regression loss $L_{box}$, classification loss $L_{cls}$, and DFL $L_{dfl}$. $\lambda_{box},\;\lambda_{cls},\;\lambda_{dfl}$ are the weight balances of each component.

Complete Intersection over Union (CIoU) Loss [37] is:

(13) $$L_{box}=1-IoU+\frac{\rho^{2}\left(b,b_{gt}\right)}{c^{2}}+\alpha v$$

(14) $$IoU=\frac{\left|B_{pred}⋂B_{gt}\right|}{\left|B_{pred}⋃B_{gt}\right|}$$

where $\rho^{2}\left(b,b_{gt}\right)$ is the Euclidean distance between the centers of predicted and ground-truth boxes, and ccc is the diagonal length of the smallest enclosing box. This term penalizes boxes that are spatially far from the ground truth. The weighting factor, Binary Cross-Entropy (BCE) classification loss, and DFL are as follows:

(15) $$\alpha =\frac{v}{1-IoU+v}$$

(16) $$L_{cls}=-\frac{1}{N}\sum_{i}\left[y_{i}\log\sigma \left({\hat{y}}_{i}\right)+\left(1-y_{i}\right)\log\left(1-\sigma ({\hat{y}}_{i})\right)\right]$$

(17) $$L_{dfl}=-[\left(\left\lceil l\right\rceil -l\right)\log p_{\left\lfloor l\right\rfloor }+\left(l-\left\lfloor l\right\rfloor \right)\log p_{\left\lceil l\right\rceil }]$$

where $y_{i}$ is the ground-truth class label (1 if present, 0 otherwise); ${\hat{y}}_{i}$ is the predicted class probability; l is the ground-truth boundary position (left, top, right, bottom); and ⌊l⌋ and ⌈l⌉ are the floor and ceiling bin indices around l. The loss is a weighted sum based on proximity to the true boundary, encouraging precise localization. In YOLO26s, l is normalized by stride, reducing the scale of DFL and stabilizing training (~0.01 vs. ~1.2 in YOLOv8).

### 2.5. Training Configuration

All training hyperparameters (Table 3) follow the Ultralytics (8.4.56) YOLO framework’s built-in default configuration: SGD momentum (0.937), weight decay (5 × 10^−4^), warm-up duration (3 epochs), and the box/classification/DFL-slot loss gains (7.5/0.5/1.5) are the framework’s documented defaults, adopted rather than tuned so that any performance difference between models reflects architecture rather than hyperparameter search. The one deliberate departure from default is the initial learning rate, lowered from the framework default of 0.01 to 1 × 10^−3^ with cosine annealing to 1 × 10^−5^, a common adjustment when fine-tuning on a dataset substantially smaller than the COCO pre-training corpus, to reduce the risk of destabilizing the pretrained weights early in training.

### 2.6. Evaluation Metrics

All models are evaluated using standard object detection metrics computed on the validation set. IoU is the basis for both the true-positive matching criterion described below and the CIoU regression loss in Equation (14). A predicted bounding box is true positive (TP) if its IoU with the correct ground-truth box is greater than 0.5 (τ = 0.5). Otherwise, it is considered a false positive (FP). This IoU threshold is separate from the confidence threshold used to decide whether a detection is reported. Unmatched ground-truth boxes are false negatives (FN):

(18) Precision (P) = TP / (TP + FP)

(19) Recall (R)=TP / (TP+FN)

Average precision (AP) summarizes the model’s precision–recall curve for a single class, and mean average precision (mAP) is the average of AP across all classes C as:

(20) $$AP=\frac{1}{100}\sum_{r\in \{0,0.01,\cdots ,1\}}\underset{r^{\prime }\geq r}{\max}P(r^{\prime })$$

(21) $$mAP=\frac{1}{C}\sum_{c=1}^{C}AP_{c}$$

where r is the recall threshold and $P(r^{\prime })$ is the precision at recall $r^{\prime }$. mAP@0.5 evaluates predicted boxes with IoU ≥ 0.5 (moderate overlap requirement). mAP@0.5:0.95 is a stricter metric, averaging AP across IoU thresholds from 0.5 to 0.95, penalizing poorly localized boxes. AP is computed via 101-point interpolated precision–recall integration, following the COCO evaluation protocol, as implemented in the Ultralytics validation pipeline used throughout this study.

The F1 score combines precision and recall into a single metric. For practical deployment, F1 is evaluated across confidence thresholds τ to select an optimal operating point:

(22) F1 = 2 × (P × R) / (P + R) 

(23) $$T^{*}=\arg\underset{T}{\max}F1(T)$$

**Figure 5:**
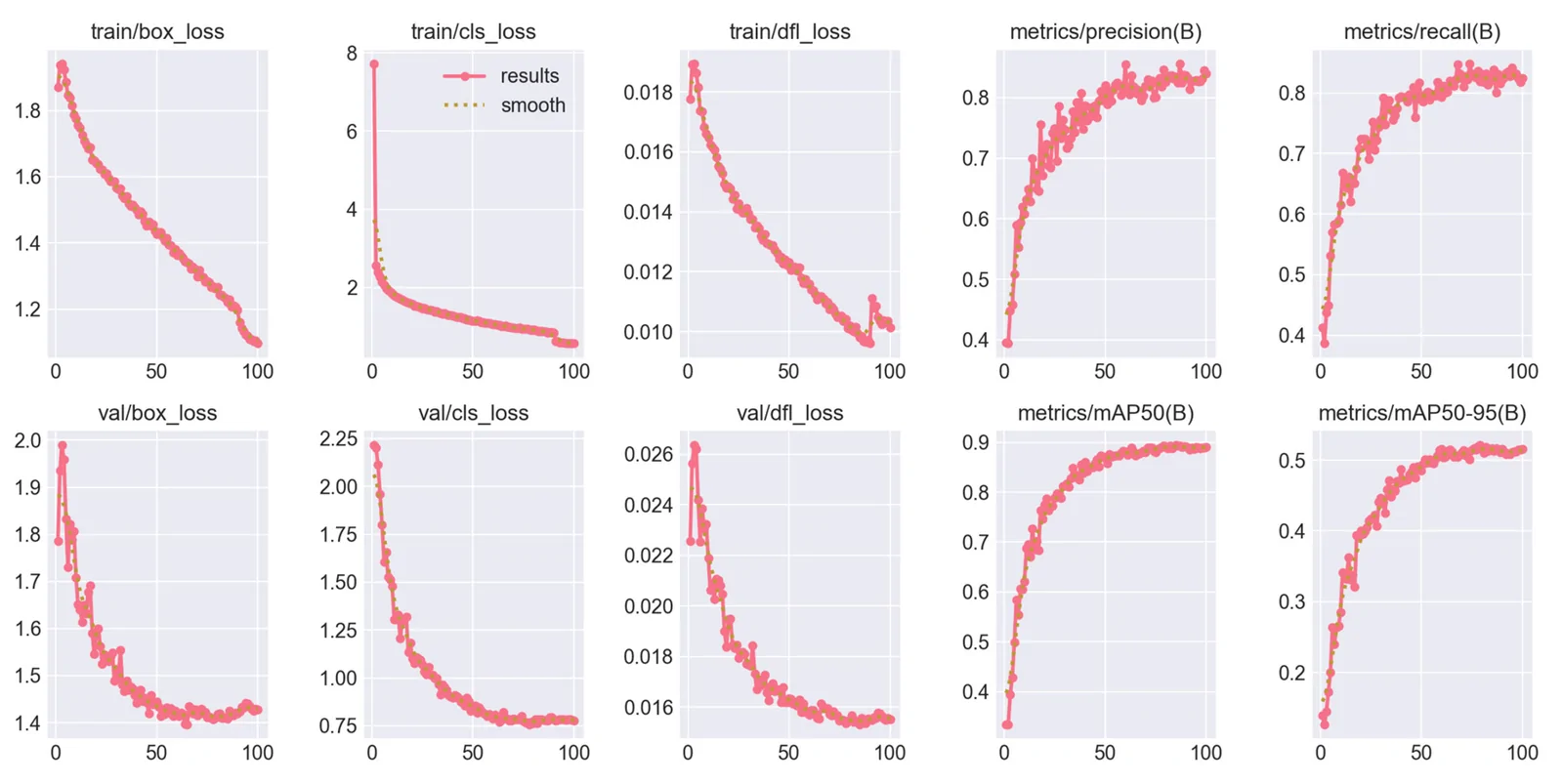
Training and validation convergence curves of YOLO26s over 100 epochs. Top row: training box, classification, and DFL-labeled regression losses. Bottom row: validation loss alongside precision, recall, and mAP@0.5.

This ensures a balance between precision and recall, critical in road maintenance applications where both false positives (unnecessary inspections) and false negatives (missed cracks) carry operational costs. Unless otherwise noted, all metrics reported in Section 3 are computed once at the end of training (epoch 100) on the full validation set; the per-epoch curves in Figure 5 track the same metrics during training solely to monitor convergence, not as a separate evaluation protocol.

## 3. Results

### 3.1. Quantitative Evaluation

The performance of YOLO26s and seven baseline models was evaluated on the 6972-image four-country RDD2022 subset using standard object detection metrics: mean average precision (mAP@0.5 and mAP@0.5:0.95), Precision, Recall, and F1 score. Table 4 summarizes the results at the final training epoch (epoch 100).

Within this single training run, YOLO26s outperforms all three baselines across every reported metric (Table 4). Table 5 shows results separately for four crack/damage types: Longitudinal Crack, Transverse Crack, Alligator Crack, and Pothole. Results are given for both YOLOv8s and YOLO26s. For both models, Alligator Crack has the highest AP@0.5 (~93%) among all classes. However, its AP@0.5:0.95 is much lower (~55%). This gap means the model’s bounding boxes are often roughly correct, but not tightly matched to the true object shape. This makes sense given alligator cracking’s irregular, sprawling pattern, which is hard to box tightly.

Repeated training across three random seeds (Table 6) confirms this ranking is directionally consistent. YOLO26s’s mean mAP@0.5 (88.52% ± 1.03%) exceeds YOLOv8s’s (87.41% ± 0.84%) by a margin comparable in magnitude to the between-seed standard deviation, so we characterize the improvement as directionally robust rather than statistically overwhelming.

### 3.2. Model Complexity and Computational Efficiency

Table 7 compares the four evaluated models in terms of parameter count, FLOPs (G), mAP@0.5, training time (hours), and weight (MB) (Table S2 with four additional baseline models). YOLO26s achieves the highest detection accuracy while using 14.3% fewer parameters and 7.7% fewer FLOPs than YOLOv8s, indicating an optimal balance between model size and performance. The smaller weight (19.2 MB) facilitates deployment on edge devices such as mobile inspection rigs or embedded systems in road maintenance vehicles, reducing energy usage and computational cost. Although training time is slightly higher due to the attention module (C2PSA), this does not affect inference speed, ensuring real-time operational feasibility.

YOLO26s achieves the highest mAP@0.5 (89.0%) while requiring 14.3% fewer parameters (9.6M vs. 11.2M) and 7.7% fewer FLOPs (26.4G vs. 28.6G) than YOLOv8s. The weight file of YOLO26s is 19.2 MB compared to 21.5 MB for YOLOv8s, a meaningful reduction for edge deployment where model storage is constrained. Training time for YOLO26s (4.01 h) slightly exceeds YOLOv8s (3.87 h) due to the additional C2PSA attention computation, but this is a one-time cost that does not affect inference latency.

### 3.3. Training Loss Convergence

Table 8 presents the final training loss values at epoch 100 for all model variants (Table S3 with four additional baseline models). All models exhibit stable, monotonically decreasing loss curves over the 100-epoch training run. It can be seen in Table 6 that the final training and validation loss components for each model are box regression loss, classification loss, and DFL. YOLO26s shows the lowest classification loss (0.5755 training, 0.7775 validation), reflecting its ability to discriminate between the four crack types effectively. Its DFL (~0.01) is significantly smaller than YOLOv8 (~1.2), illustrating the effect of stride-normalized DFL for precise bounding-box localization. The narrow gap between training and validation losses indicates effective regularization through data augmentation and confirms that YOLO26s generalizes well to unseen data.

Figure 5 shows the evolution of training and validation losses over 100 epochs. The top row shows box loss, classification loss, and DFL on the training set, while the bottom row depicts validation losses alongside precision, recall, and mAP metrics. All losses decrease monotonically, and precision, recall, and mAP steadily increase, demonstrating stable optimization and effective learning. Simultaneously, precision, recall, and mAP metrics steadily improve before reaching saturation near the final epochs, demonstrating strong detection accuracy and generalization performance. The final performance approaches approximately 89.0% mAP@0.5 and 51.6% mAP@0.5:0.95, confirming the effectiveness of YOLO26s for multi-class road damage detection.

### 3.4. Confidence-Based Evaluation

**Figure 6:**
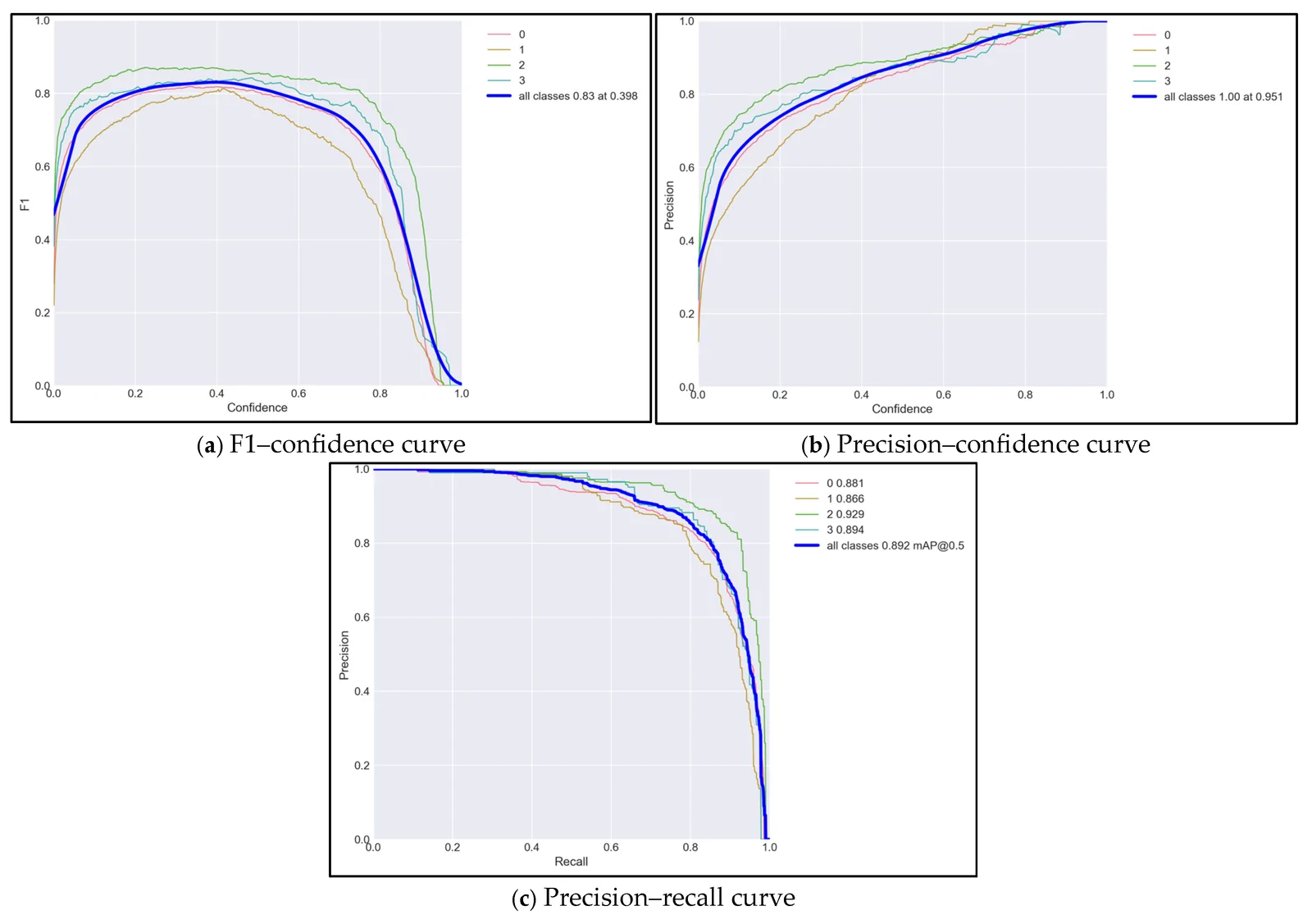
Validation performance analysis of YOLO26s. (**a**) F1–confidence curves for four crack classes, (**b**) precision–confidence curve, (**c**) precision–recall curves with per-class AP and overall mAP@0.5.

To assess the robustness and reliability of YOLO26s across different confidence thresholds and crack types, we conducted a comprehensive validation analysis. Figure 6 shows the validation performance analysis of YOLO26s. Figure 6a: F1–confidence curves for longitudinal, transverse, pothole, and alligator cracks, showing how F1 score varies with detection confidence; (b) precision–confidence curve depicting the relationship between predicted confidence and precision across all crack classes; and (c) per-class precision–recall (PR) curves along with the overall mean average precision (mAP@0.5). These visualizations collectively provide insights into model calibration, optimal operating thresholds, and the relative difficulty of detecting each crack type.

It can be seen in Figure 6 that the overall F1 score peaks at approximately 0.83 for a confidence threshold near 0.398, indicating the threshold that balances precision and recall optimally. Class-wise analysis reveals that pothole cracks are detected with the highest F1 over a broad range, while transverse and alligator cracks are slightly more challenging, highlighting morphological variability. Part (b) demonstrates that precision increases monotonically with confidence threshold, starting at 0.20 for τ = 0.1 and peaking at 0.68 for τ = 1.0. This confirms that higher-confidence predictions reduce false positives while maintaining recall, which is critical for reliable automated inspections. The smooth decline in precision with increasing recall indicates balanced detection performance, and the overall mAP@0.5 of 0.892 confirms robust multi-class detection across all crack types.

### 3.5. Confusion Matrix Analysis

**Figure 7:**
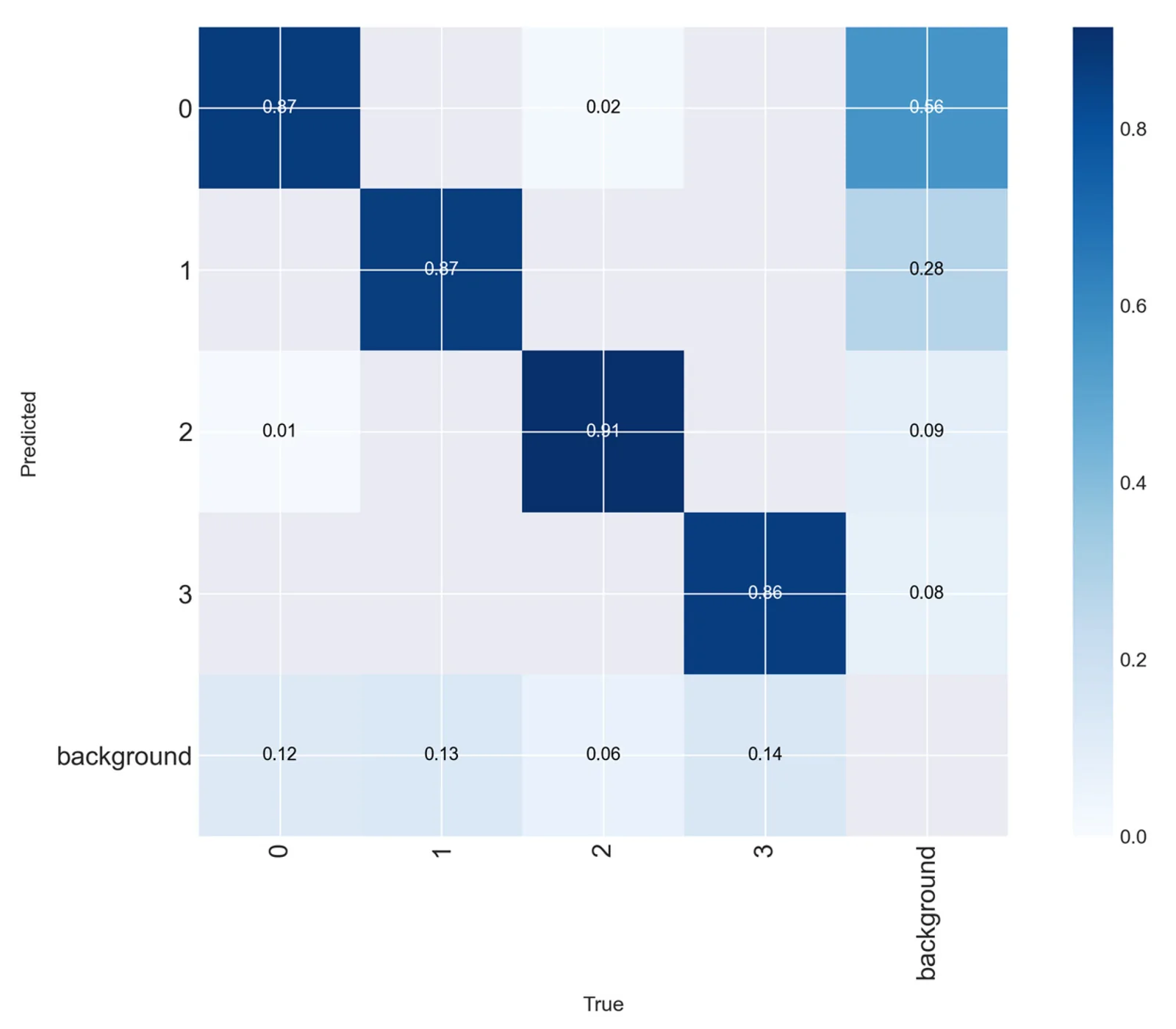
Normalized confusion matrix for the road damage detection model (YOLO26s) evaluated on the validation set. Values represent the proportion of true instances of each class that are predicted as each class or as background.

Figure 7 shows the normalized confusion matrix for YOLO26s on the validation set, constructed at a confidence threshold of 0.25 and an IoU matching threshold of 0.45 (Ultralytics default validation settings). In the matrix, rows represent predicted classes, columns represent true (ground-truth) classes, and the bottom row/rightmost column represent, respectively, true instances the model failed to detect (false negatives) and predictions with no corresponding true object (false positives). Diagonal accuracy exceeds 0.85 for all four classes (longitudinal = 87.3%, transverse = 86.7%, alligator = 91.5%, pothole = 85.8%). The largest cross-class confusion is between Longitudinal and Alligator cracks (6–11 instances in each direction, out of hundreds of correct detections per class), not between any other class pair. Row-normalized false-positive rates show how often a model’s predictions were wrong. Specifically, they show the fraction of predictions for a class where the true label was actually background (no object). For YOLO26s, these rates range from 13.2% (alligator) to 28.7% (transverse). For YOLOv8s, they range from 20.7% to 36.2%, across the same four classes. YOLO26s has lower false-positive rates than YOLOv8s for all four classes. This means YOLO26s produces fewer false alarms than YOLOv8s, at the same operating thresholds. This result matches YOLO26s’s higher overall Precision score (Table 4). Computational efficiency is addressed separately via the deployment benchmarking in Section 4.3.

### 3.6. Qualitative Detection Results

**Figure 8:**
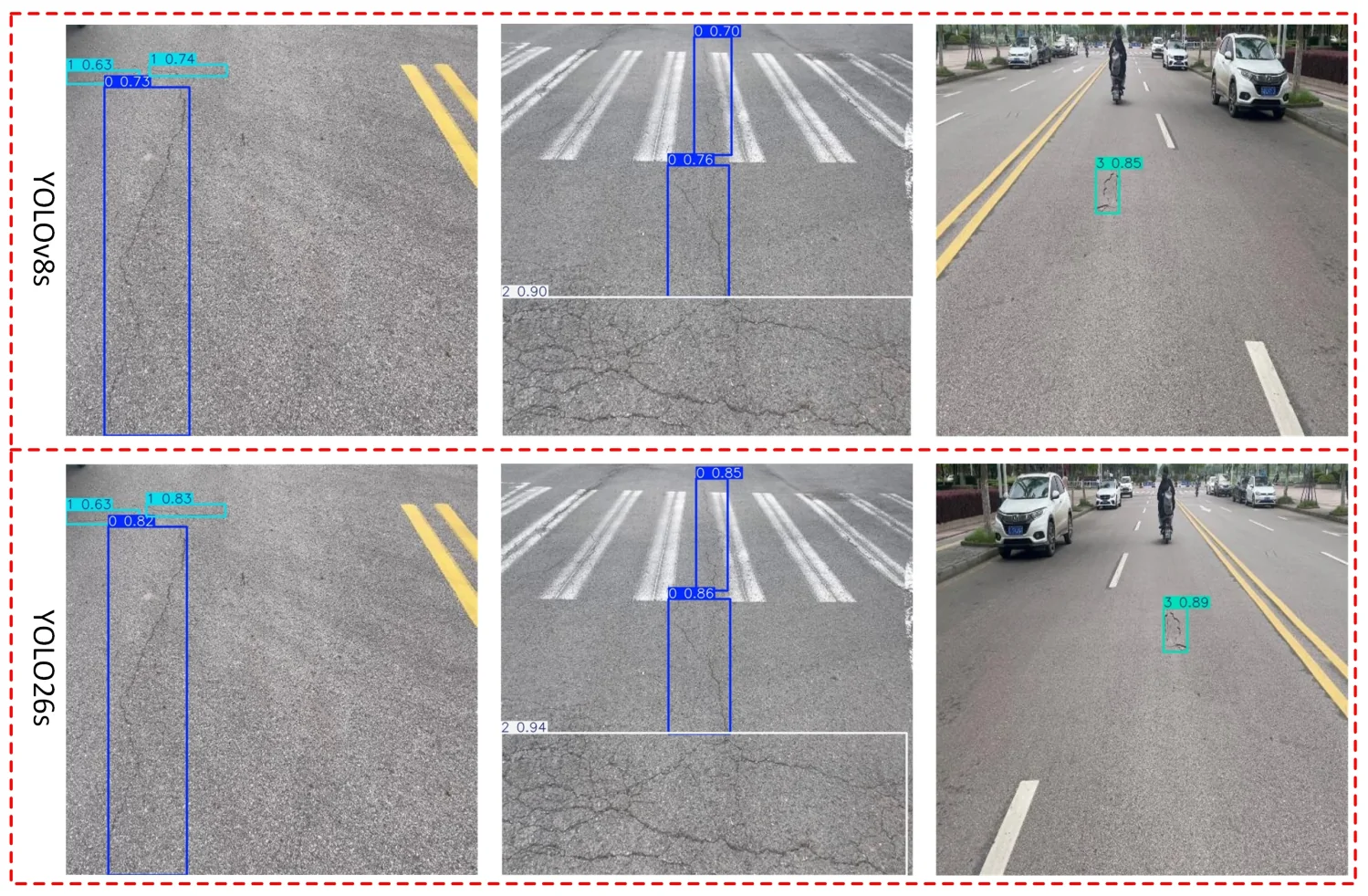
Qualitative detection comparison between YOLOv8s (**top row**) and YOLO26s (**bottom row**) on three representative road inspection scenes.

Qualitative inspection of YOLO26s detection outputs confirms accurate localization of crack regions across diverse road surfaces and illumination conditions. The model demonstrates particular strength in detecting alligator cracks, which exhibit complex, non-uniform morphology and variable spatial extent. YOLO26s also maintains high recall for thin longitudinal and transverse cracks, challenging detection targets due to their narrow pixel footprint and low contrast against certain pavement backgrounds. Figure 8 compares YOLO26s and YOLOv8s predictions on representative images. YOLO26s provides higher-confidence scores for the same cracks and more precise bounding boxes, particularly for longitudinal and transverse cracks. This demonstrates improved discriminative capability for complex and irregular crack morphologies.

Figure 8 compares YOLO26s and YOLOv8s predictions on sample images. YOLO26s produces higher-confidence scores for identical cracks and more precise bounding-boxes, especially for longitudinal and transverse cracks (class 0: 0.82 vs. 0.73), transverse cracks (class 0: 0.86 vs. 0.76; class 2: 0.94 vs. 0.90), and a non-pavement object (class 3: 0.89 vs. 0.85) while maintaining comparable bounding-box localization, demonstrating its stronger discriminative capability without sacrificing detection coverage. The figure confirms the model’s improved discriminative capability and localization accuracy, critical for reducing false alerts and optimizing repair schedules, thus enhancing the environmental and operational efficiency of road maintenance.

## 4. Discussion

### 4.1. Comparison with State-of-the-Art Methods

To contextualize the performance of YOLO26s within the broader literature on deep learning-based road damage detection, Table 9 presents a consolidated comparison within the study and against eleven published methods alongside our four evaluated models. The eleven external methods were evaluated on different datasets, primarily the full RDD2022 benchmark [23], a US-only subset of RDD2022 [38], UAV-collected crack imagery [39,40], or independently collected pavement datasets and consequently their absolute metric values are not directly numerically comparable to our results, which are obtained on a curated four-country subset of RDD2022 comprising 6972 images. The comparison is included to provide architectural context and to situate YOLO26s within the evolutionary trajectory of YOLO-based road damage detection. Direct same-dataset comparisons are confined to the four models trained in this study. Only the four models evaluated in this study are trained under identical conditions and are therefore directly comparable to one another; the table situates them within the broader literature for architectural context only.

The subset was obtained via Roboflow [23] in YOLO annotation format and is fully derived from the original RDD2022 benchmark; it is not a privately collected custom dataset. “RDD2022 US” refers to the US-only subset used by external studies [38]. Rows marked with “†” are evaluated on different datasets and should not be interpreted as direct performance comparisons. Within-study comparison (same dataset): among the four models evaluated on the same RDD2022 four-country subset, YOLO26s consistently achieves the highest performance across all five reported metrics. It attains a mAP@0.5 of 89.0%, surpassing the scale-equivalent YOLOv8s baseline by +0.9 percentage points, and a mAP@0.5:0.95 of 51.6%, outperforming YOLOv8s by +2.1 pp, a meaningful gain on the stricter COCO-standard metric that penalizes imprecise bounding-box fits. YOLO26s simultaneously achieves these accuracy gains with 14.3% fewer parameters (9.6M vs. 11.2M) and a 10.6% smaller weight file (19.2 MB vs. 21.5 MB), confirming a favorable accuracy–efficiency trade-off. YOLO26n, while the most compact model at 5.2 MB and 2.6M parameters, trails YOLO26s by 3.3 pp in mAP@0.5, which, for safety-critical road maintenance applications, justifies YOLO26s as the more favorable choice on GPU hardware, though not uniformly faster across all evaluated hardware, among the four models evaluated for the deployment scenario targeted in this study.

Cross-study architectural context: among external methods evaluated on the full RDD2022 benchmark, the YOLO-family baseline models serve as useful reference points. The YOLOv8n and YOLOv8s base configurations evaluated by Zeng and Zhong [46] on the full 47,420-image RDD2022 dataset report mAP@0.5 values of 78.6% and 80.5% respectively, notably lower than the 86.4% and 88.1% achieved by the same architectures in our study. This difference is expected: our four-country subset of 6972 images is structurally more homogeneous and is trained with COCO-pretrained weights applied to a narrower geographic and climatic distribution, yielding higher absolute mAP on that specific subset. This highlights the importance of reporting dataset provenance alongside benchmark values, as cross-dataset comparisons without this context can be misleading.

The RDD-YOLO model [45], such as YOLOv8x with SimAM and bilinear upsampling trained on RDD2022, achieves 62.5% mAP@0.5, which is low relative to our models due to the more challenging full validation set (six countries, greater geographic variation, and class imbalance). Similarly, YOLOv8-PD [46] reports incremental improvements over YOLOv8n (+4.1 pp mAP@0.5, +2.1 pp mAP@0.5:0.95) rather than absolute values for the road damage generalization split, precluding a direct numeric comparison. YOLO11-MBC [47] and UAV-YOLOv8 [48] operate on entirely different domains (single-location road and UAV aerial imagery respectively), preventing meaningful metric comparison. YOLOv8-DGS [49] reports the highest precision (91.6%) and mAP@0.5 (92.4%) in the table. These results are obtained on a self-collected, single-location pavement dataset used for intelligent joint-filling equipment, which is structurally simpler than a multinational dataset: it contains fewer environmental conditions, a more uniform camera angle, and controlled lighting.

Collectively, the evidence in Table 9 supports the conclusion that YOLO26s achieves a competitive accuracy–efficiency ratio for multinational pavement damage detection, outperforming equivalent-scale YOLOv8 baselines on the same dataset while requiring fewer computational resources. The performance superiority of YOLO26s can be attributed to three complementary architectural advantages. This reflects the combined benefit of more capacity-efficient backbone representations and attention-guided feature selection. Within-study comparison (same dataset): among the four models evaluated on the same RDD2022 four-country subset, YOLO26s achieves the highest value on all five reported metrics in this single training run (Table 4), a ranking that repeated-seed training (Table 5) confirms is directionally consistent though not large relative to seed-to-seed variability.

### 4.2. Cross-Architecture Comparison: YOLO26s vs. YOLOv8s

**Figure 9:**
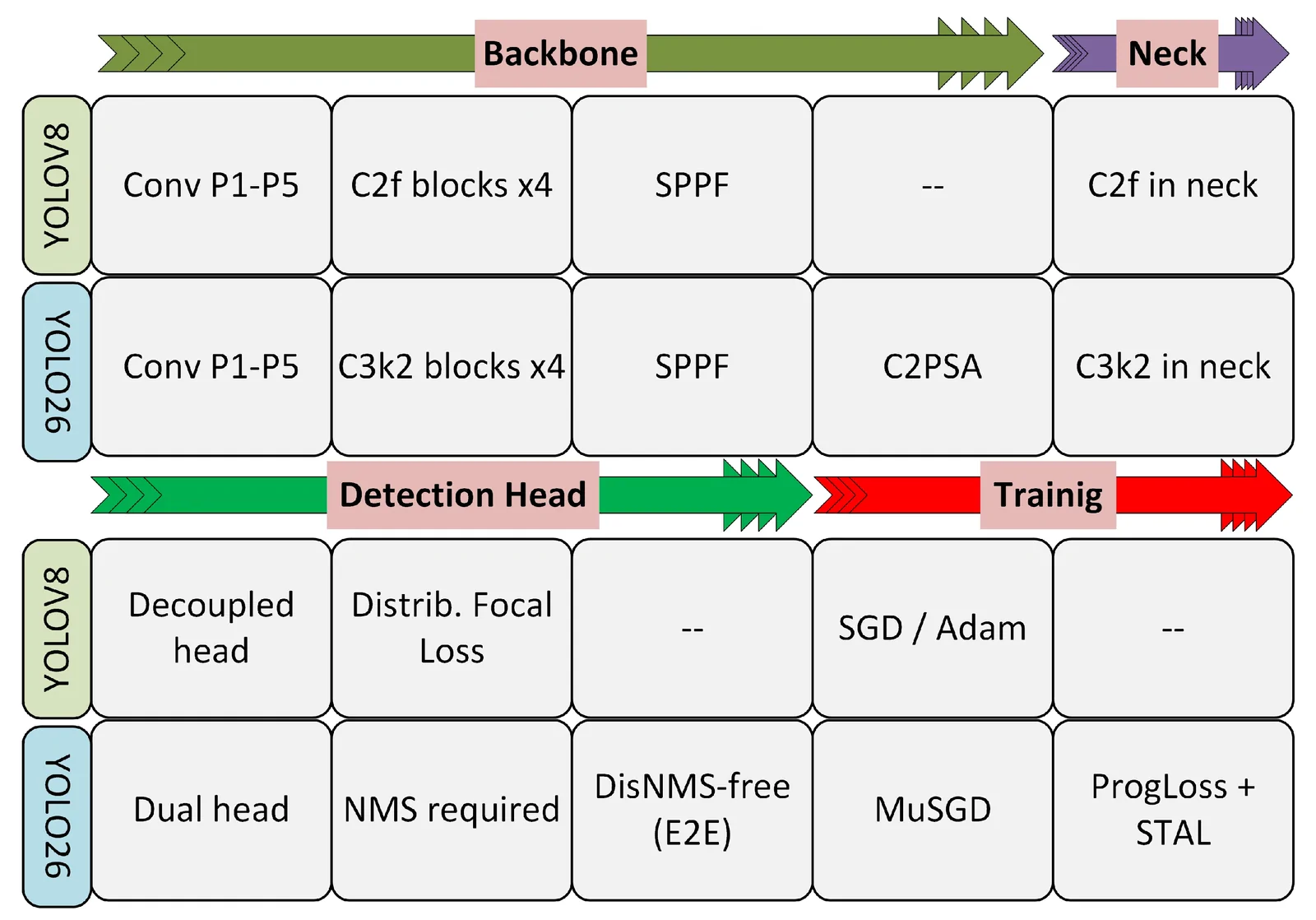
Architectural comparison of YOLOv8 and YOLO26 across four pipeline stages, such as backbone, neck, detection head, and training. Double dash indicates a feature absent from that model at that stage.

The YOLO26s versus YOLOv8s comparison is the most informative in this study because it isolates the impact of architectural evolution at equivalent model scale, as shown in Figure 9. YOLO26s delivers higher accuracy (+0.9 pp mAP@0.5, +2.1 pp mAP@0.5:0.95) with a smaller model and shorter inference graph. YOLO26s processes more images per second than YOLOv8s at the same resolution while detecting cracks more precisely—a key advantage for real-time road inspection vehicles.

Figure 9 is divided into two horizontal bands. The top band covers the backbone and neck stages, marked by a large olive-green arrow for the backbone and a smaller purple arrow for the neck. Two changes are most directly implicated in the accuracy gain reported in Table 4, and we now have direct ablation evidence to weigh them rather than relying on architectural rationale alone. We trained a YOLO26s variant with the C2PSA module surgically removed, while holding hyperparameters, data, and pretrained initialization identical to the full model (Table 10).

Removing C2PSA caused a large drop in performance, reducing mAP@0.5 by 11.51 percentage points (89.25% to 77.74%) and mAP@0.5:0.95 by 10.94 percentage points. This effect is far larger than the +0.9 to +2.1 percentage point advantage that full YOLO26s has over YOLOv8s. This indicates that C2PSA, rather than the C3k2 backbone or the DFL-free head (Section 2.2.3), is the dominant architectural driver of YOLO26s’s performance on this task, plausibly because pavement cracks are thin, low contrast, and spatially sparse targets that benefit disproportionately from attention-guided feature selection relative to the larger, more homogeneous objects typical of general-purpose detection benchmarks. The DFL-free head is instead consistent with the inference-latency advantage reported in Section 4.3 rather than with accuracy directly.

### 4.3. Deployment Benchmarking and Practical Implications

In this study, we measured inference latency directly rather than relying on parameter count and FLOPs alone. On a cloud GPU (NVIDIA GeForce RTX 5070, via Google Colab) and a genuine embedded device, a Raspberry Pi (via ONNX export), as shown in Table 11, YOLO26s is faster than YOLOv8s on both devices. On the GPU, it runs at 22.77 ms per image vs. 24.21 ms for YOLOv8s. On the Raspberry Pi, it runs at 364.81 ms per image vs. 418.18 ms for YOLOv8s. These direct measurements support the claim of real-time edge deployment, rather than relying only on parameter count and FLOPs.

We also evaluated both models on synthetically degraded validation images simulating rain, fog, low light, motion blur, and shadow (Table 12). Both models are highly vulnerable to motion blur specifically. mAP@0.5 drops from a clean baseline of 88.6–89.3% to approximately 15–16% (a 72–74 percentage point collapse), consistent with motion blur disproportionately destroying the thin, low-contrast crack features both models rely on. Fog and low light produce moderate degradation (16–21 percentage point drops), while rain and shadow are comparatively mild (2–3 percentage points). Neither model is uniformly more robust: YOLO26s degrades less under low light and shadow, but very slightly more under fog and motion blur, than YOLOv8s. Three properties of YOLO26s are directly relevant to field deployment. First, its smaller weight file (19.2MB vs. 21.5MB for YOLOv8s) reduces storage and over-the-air update costs across a fleet of inspection vehicles or fixed roadside cameras. Second, the measured GPU inference latency above confirms real-time operation is achievable rather than merely inferred from FLOPs, pending the CPU/embedded confirmation noted above. Third, YOLO26 is natively NMS-free. This means its speed does not drop in scenes with many overlapping detections. This matters for densely alligator-cracked pavement, which often creates many overlapping candidate boxes under YOLOv8′s NMS-based system.

### 4.4. Multinational Dataset Impact

The use of a multinational RDD2022 subset introduces complexity not present in single-country studies. Norway’s road imagery includes snow-covered pavement and low-angle winter lighting; Japan’s urban imagery includes narrow streets with lane markings that may be confused with longitudinal cracks; China’s motorbike camera imagery has a distinctively low camera angle and higher motion blur than dashcam footage; and United States imagery features wide lanes and high-contrast pavement markings. Table 13 shows mAP@0.5, mAP@0.5:0.95, Precision, Recall, and F1 for both models.

YOLO26s outperforms YOLOv8s in three of the four countries (Japan: 84.38% vs. 82.76% mAP@0.5; Norway: 83.08% vs. 80.43%; United States: 78.88% vs. 73.36%), with China essentially tied (97.80% vs. 97.91%, both near ceiling). China is markedly easier for both models than the other three countries, plausibly reflecting more consistent camera angle and lighting in the China_MotorBike imagery relative to the more variable dashcam conditions elsewhere. The pattern is that YOLO26s’s largest advantages occur in the harder cases, which directly demonstrates the attention module’s substantial contribution to overall performance, rather than relying on architectural motivation alone.

### 4.5. Limitations and Future Work

We organize the following as a prioritized roadmap directly tied to this study’s central objective: practical, real-time edge deployment of multi-class crack detection. (1) Deployment validation (partially addressed): GPU inference latency was measured directly (Section 4.3); CPU-laptop and Raspberry Pi measurements remain the concrete near-term next step. (2) Robustness to adverse conditions (addressed): synthetic rain, fog, low-light, motion-blur, and shadow evaluation (Section 4.3) shows both models are highly vulnerable to motion blur specifically (−72 to −74 percentage points mAP@0.5) and moderately vulnerable to fog and low light, with neither model uniformly superior across all five conditions; real (not synthetic) adverse-weather imagery collection remains future work. (3) Domain adaptation: fine-tuning on country-specific subsets to quantify the benefit of local adaptation versus the multinational model is evaluated here. (4) Statistical robustness (addressed): repeated-seed training (Section 3.1) confirms the YOLO26s over YOLOv8s ranking is directionally consistent, though the margin is comparable in size to seed-to-seed variability. (5) Component-level ablation (addressed): isolating C2PSA’s contribution (Section 4.2) shows it is the dominant architectural driver of YOLO26s’s performance gain, substantially larger than the C3k2 or DFL-free-head effects. (6) Severity quantification combines bounding boxes with pixel-wise crack-width estimation, INT8 quantization, WIoU vs. CIoU loss comparison, and extension to video streams for continuous monitoring.

## 5. Conclusions

This study presents YOLO26s, a lightweight DL model for multi-class pavement crack detection, evaluated on a curated four-country subset of the RDD2022 (6972 images). Across all quantitative metrics, YOLO26s outperforms baseline models (YOLOv8s, YOLOv8n, YOLO26n), achieving mAP@0.5 of 89.0% and mAP@0.5:0.95 of 51.6%, while reducing parameters and FLOPs, making it suitable for real-time edge deployment. The proposed approach enables accurate detection of longitudinal, transverse, pothole, and alligator cracks under diverse environmental conditions and road textures. Qualitative results and analyses confirm robust multi-class discrimination and minimal false positives. Confidence-threshold evaluation further identifies optimal operating points for practical deployment, balancing precision and recall. The findings demonstrate that YOLO26s can facilitate proactive maintenance planning, reduce material and energy consumption, and support sustainable infrastructure management. Future work will extend the framework to video-based monitoring, severity quantification, domain-specific fine-tuning, and INT8 quantization for enhanced edge efficiency. Overall, YOLO26s represents a step toward sustainable, AI-driven pavement management, combining high detection accuracy, operational efficiency, and environmental benefits.

## Figures and Tables

**Table 1:** Road damage class definitions.

Class ID	Category	Description
0	Longitudinal Crack	Cracks parallel to the road centerline, caused by traffic loading and subgrade settlement.
1	Transverse Crack	Cracks perpendicular to road direction, caused by thermal expansion/contraction and shrinkage.
2	Pothole	Rectangular network of cracks dividing the surface into potholes, caused by volume change and aging.
3	Alligator Crack	Interconnected fatigue crack network resembling alligator skin, indicating deep structural failure.

**Table 2:** Training hyperparameters.

Models	Parameters	FLOPs (G)	Notes
YOLOv8s	11.2M	28.6	Small-scale YOLOv8
YOLOv8n	3.2M	8.7	Nano-scale YOLOv8
YOLO26n	2.6M	6.9	Nano-scale YOLO26
This work	9.6M	26.4	Proposed model

**Table 3:** Training hyperparameters applied uniformly across YOLO26s and all baseline models.

Hyperparameter	Value
Input Resolution	640 × 640 pixels
Epochs	100
Batch Size	16
Optimizer	SGD with momentum
Initial Learning Rate	1 × 10^−3^
Learning Rate Schedule	Cosine annealing to 1 × 10^−5^
Warmup Epochs	3
Weight Decay	5 × 10^−4^
Momentum	0.937
Loss Weights (λ_box/λ_cls/λ_dfl)	7.5/0.5/1.5
Pre-training	COCO-pretrained weight

**Table 4:** Final validation metrics (Epoch 100).

Models	mAP@0.5	mAP@0.5:0.95	Precision	Recall	F1 Score
YOLOv8n	86.4%	48.9%	80.6%	81.7%	81.2%
YOLOv8s	88.1%	49.5%	82.4%	82.3%	82.4%
YOLO11n	87.6%	50.1%	82.2%	79.5%	80.8%
YOLO11s	88.6%	51.6%	82.2%	81.8%	82.0%
YOLO12n	87.9%	50.5%	81.6%	81.5%	81.6%
YOLO12s	89.2%	52.2%	83.9%	81.4%	82.9%
YOLO26n	85.7%	49.0%	80.9%	79.4%	80.1%
This work	89.0%	51.6%	84.0%	82.5%	83.2%

**Table 5:** Per-class performance comparison for YOLOv8s and YOLO26s.

Model	Class	Precision	Recall	AP@0.5	AP@0.5:0.95	F1
YOLOv8s	Longitudinal Crack	82.64%	82.32%	88.59%	54.88%	82.48%
YOLOv8s	Transverse Crack	79.48%	74.17%	83.26%	46.29%	76.73%
YOLOv8s	Alligator Crack	85.53%	88.10%	93.44%	55.34%	86.79%
YOLOv8s	Pothole	78.50%	82.95%	89.27%	45.59%	80.66%
YOLOv8s	ALL (mean)	81.53%	81.88%	88.64%	50.53%	81.67%
YOLO26s	Longitudinal Crack	82.41%	80.97%	88.06%	55.37%	81.69%
YOLO26s	Transverse Crack	81.91%	79.23%	86.61%	50.17%	80.55%
YOLO26s	Alligator Crack	88.41%	85.58%	92.88%	55.90%	86.97%
YOLO26s	Pothole	84.82%	82.39%	89.47%	47.42%	83.59%
YOLO26s	ALL (mean)	84.39%	82.04%	89.25%	52.22%	83.20%

**Table 6:** Seed variability.

Model	mAP@0.5 (Mean ± Std)	mAP@0.5:0.95 (Mean ± Std)	Precision (Mean ± Std)	Recall (Mean ± Std)	F1 (Mean ± Std)
YOLOv8s	87.41% ± 0.84%	49.89% ± 0.18%	82.21% ± 0.68%	80.90% ± 1.78%	81.67% ± 0.66%
YOLO26s	88.52% ± 1.03%	51.06% ± 0.27%	83.12% ± 1.43%	81.58% ± 1.26%	82.40% ± 1.08%

**Table 7:** Model complexity comparison.

Models	Parameters	FLOPs (G)	mAP@0.5	Training Time	Weight (MB)
YOLOv8n	3.2M	8.7	86.4%	2.71	6.2
YOLOv8s	11.2M	28.6	88.1%	3.87	21.5
YOLO26n	2.6M	6.9	85.7%	2.67	5.2
This work	9.6M	26.4	89.0%	4.01	19.2

**Table 8:** Training and validation loss (epoch 100).

Models	Box Loss (Train)	Cls Loss (Train)	DFL (Train)	Cls Loss (Val)
YOLOv8n	1.1386	0.7930	1.2048	0.8834
YOLOv8s	0.9715	0.6240	1.1298	0.8539
YOLO26n	1.2072	0.7097	0.0112	0.8642
This work	1.0958	0.5755	0.0101	0.7775

**Table 9:** Comparison with state-of-the-art methods and within study.

Models	Datasets	P (%)	R (%)	F1 (%)	mAP@0.5 (%)	mAP@0.5:0.95 (%)	Weight (MB)
Faster R-CNN [41] †	RDD2022 (a)	68.3	61.4	64.7	63.5	34.2	~160
YOLOv5s [42] †	RDD2022 US (c)	73.0	71. 0	72.0	75.0	49.0	14.1
YOLOv7 [43] †	RDD2022 (a)	71.5	69.3	70.4	73.1	47.3	71.3
YOLOv8n base [44] †	RDD2022 (a)	79.4	77.2	78.3	78.6	55.8	6.2
YOLOv8s base [44] †	RDD2022	81.1	79.8	80.4	80.5	57.4	21.5
RDD-YOLO [45] †	RDD2022	—	—	—	62.5	36.4	~22
YOLOv8-PD [46] †	RDD2022 (b)	—	—	+0.5	+4.1 over base	+2.1 over base	~8.9
YOLO11 [38] †	RDD2022 US (c)	83.8	79.7	81.7	87.8	61.2	18.4
YOLO11-MBC [47] †	Custom	61.0	70.0	72.0	75.0	66.0	~19
UAV-YOLOv8 [48] †	UAV-Crack	75.6	76.4	75.3	79.3	—	~22
YOLOv8-DGS [49] †	Custom (d)	91.6	90.0	90.8	92.4	—	~22
YOLOv8n (ours)	RDD2022-sub, N = 6972	80.6	81.7	81.2	86.4	48.9	6.2
YOLOv8s (ours)	RDD2022-sub, N = 6972	82.4	82.3	82.4	88.1	49.5	21.5
YOLO26n (ours)	RDD2022-sub, N = 6972	80.9	79.4	80.1	85.7	49.0	5.2
YOLO26s (ours)	RDD2022-sub, N = 6972	84.0	82.5	83.2	89.0	51.6	19.2

Note: Only the four models evaluated in this study (YOLOv8n, YOLOv8s, YOLO26n, YOLO26s) are trained and evaluated on the identical dataset, split, and hyperparameters, and are therefore directly comparable. † Metrics as published; (a) full RDD2022; (b) generalization split; (c) US subset; (d) custom single-location dataset.

**Table 10:** C2PSA ablation.

Model	Params	GFLOPs	mAP@0.5	mAP@0.5:0.95	Precision	Recall
YOLO26s (full)	9.6M	26.4	89.25%	52.22%	84.39%	82.04%
YOLO26s (no C2PSA)	9.0M	21.7	77.74%	41.28%	71.10%	71.46%
Difference	−0.6M	−4.7	−11.51 pp	−10.94 pp	−13.29 pp	−10.58 pp

**Table 11:** Inference latency and throughput across deployment targets.

Device	Model	Mean ms/Image	Std (ms)	FPS
Cloud GPU NVIDIA GeForce RTX 5070	YOLOv8s	24.21	24.12	41.31
Cloud GPU NVIDIA GeForce RTX 5070	YOLO26s	22.77	4.86	43.92
Raspberry Pi 5 8gb RAM	YOLOv8s	418.18	1.22	2.39
Raspberry Pi 5 8gb RAM	YOLO26s	364.81	1.30	2.74

**Table 12:** Model performance under synthetically degraded image conditions.

Model	Condition	mAP@0.5	mAP@0.5:0.95	Precision	Recall	Drop vs. Clean (mAP@0.5, pp)
YOLOv8s	clean	88.64%	50.53%	81.53%	81.88%	0.0
YOLOv8s	rain	86.55%	48.82%	82.04%	79.72%	2.09
YOLOv8s	fog	68.47%	35.58%	71.79%	62.23%	20.17
YOLOv8s	low-light	70.47%	38.59%	77.09%	64.34%	18.17
YOLOv8s	motion-blur	16.21%	6.12%	50.69%	17.52%	72.43
YOLOv8s	shadow	85.53%	48.02%	78.93%	80.03%	3.11
YOLO26s	clean	89.25%	52.22%	84.39%	82.04%	0.0
YOLO26s	rain	86.95%	50.13%	82.43%	80.11%	2.30
YOLO26s	fog	68.12%	35.52%	77.11%	59.26%	21.13
YOLO26s	low-light	73.12%	40.89%	78.31%	65.57%	16.13
YOLO26s	motion-blur	15.30%	6.38%	46.12%	17.19%	73.95
YOLO26s	shadow	86.59%	49.34%	82.87%	77.63%	2.66

**Table 13:** Per-country performance comparison of YOLOv8s and YOLO26s.

Country	Model	N Images	Precision	Recall	mAP@0.5	mAP@0.5:0.95	F1
China	YOLOv8s	228	94.55%	95.76%	97.91%	68.27%	95.14%
China	YOLO26s	228	95.43%	94.38%	97.80%	70.59%	94.90%
Japan	YOLOv8s	283	78.02%	75.58%	82.76%	40.00%	76.69%
Japan	YOLO26s	283	78.29%	79.34%	84.38%	43.48%	78.77%
Norway	YOLOv8s	140	77.19%	73.90%	80.43%	37.52%	75.46%
Norway	YOLO26s	140	88.67%	72.73%	83.08%	41.62%	79.75%
United States	YOLOv8s	449	69.22%	67.48%	73.36%	41.19%	68.15%
United States	YOLO26s	449	75.27%	72.67%	78.88%	42.79%	73.76%

## Data Availability

The datasets generated and/or analyzed during the current study are available at https://github.com/Saifal-abbas/YOLO26s-for-Multi-Class-Pavement-Crack-Detection (accessed on 22 June 2026).

## Supplementary Materials

The following supporting information can be downloaded at: https://www.mdpi.com/article/10.3390/s26165113/s1, Table S1: Training hyperparameters; Table S2: Model complexity comparison; Table S3: Training and Validation Loss, Epoch 100.
