# Supplementary material for: Evaluating YOLO26s for Multi-Class Pavement Crack Detection: A Lightweight Approach for Sustainable Edge Deployment
Source: Sensors (Basel). 2026 Aug 12;26(16):5113. doi: 10.3390/s26165113 (PMC13517557; doi:10.3390/s26165113)
Supplement: Supplementary file 1 [file sensors-26-05113-s001.zip › sensors-4424309-supplementary.pdf]

Supplementary File

With four additional baseline models

Table S1: Training hyperparameters

Model	Parameters	FLOPs (G)	Notes
YOLOv8s	11.2M	28.6	Small-scale YOLOv8
YOLOv8n	3.2M	8.7	Nano-scale YOLOv8
YOLO11n	2.58M	6.44	Nano-scale YOLO11
YOLO11s	9.41M	21.55	Small-scale YOLO11
YOLO12n	2.56M	6.48	Nano-scale YOLO12
YOLO12s	9.23M	21.53	Small-scale YOLO12
YOLO26n	2.6M	6.9	Nano-scale YOLO26
YOLO26s	9.6M	26.4	Proposed model

Table S2: Model complexity comparison

Model	Parameters	FLOPs (G)	mAP@0.5	Training Time (h)	Weight (MB)
YOLOv8n	3.2M	8.7	86.4%	2.71	6.2
YOLOv8s	11.2M	28.6	88.1%	3.87	21.5
YOLO11n	2.5M	6.4	87.7%	1.66	5.2
YOLO11s	9.4M	21.5	88.5%	2.25	20.3
YOLO12n	2.5M	6.4	88.0%	2.17	5.3
YOLO12s	9.2M	21.5	89.2%	3.23	19.5
YOLO26n	2.6M	6.9	85.7%	2.67	5.2
YOLO26s (this work)	9.6M	26.4	89.0%	4.01	19.2

Table S3: Training and Validation Loss, Epoch 100

Model	Box Loss (train)	Cls Loss (train)	DFL Loss (train)	Cls Loss (val)
YOLOv8n	1.1386	0.7930	1.2048	0.8834
YOLOv8s	0.9715	0.6240	1.1298	0.8539
YOLO11n	1.1784	0.9584	1.1734	1.0572
YOLO11s	0.9181	0.5418	1.0341	0.8278
YOLO12n	1.0635	0.8161	1.1327	1.0342
YOLO12s	0.8287	0.4630	1.0209	0.8398
YOLO26n	1.2072	0.7097	0.0112	0.8642
YOLO26s (this work)	1.0958	0.5755	0.0101	0.7775
